# Case report: cardiac tamponade secondary to candida pericarditis

**DOI:** 10.1093/jscr/rjae150

**Published:** 2024-03-15

**Authors:** Nicholas Jette, Colin Gebhardt, Dimitri Coustinos

**Affiliations:** Department of Surgery, University of Saskatchewan, Saskatoon, SK S7N 0W8, Canada; Department of Internal medicine, University of Saskatchewan, Saskatoon, SK S7N 0W8, Canada; Intensive Care, University of Saskatchewan, Saskatoon, SK S7N 0W8, Canada; Department of Thoracic Surgery, University of Saskatchewan, Saskatoon, SK S7N 0W8, Canada

**Keywords:** fungal pericarditis, fungemia, pericardial tamponade

## Abstract

Fungal pericarditis, a rare clinical presentation primarily observed in post-cardiothoracic surgery and immunocompromised patients, requires prompt recognition and effective treatment involving antifungal medications and surgical drainage. We report the case of a 40-year-old female initially diagnosed with infective endocarditis who progressed to cardiac tamponade. Timely surgical drainage significantly improved the patient’s clinical status and revealed fungal pericarditis through pathological analysis. This case highlights the importance of considering the diagnosis of fungal pericarditis even in the absence of prior cardiothoracic surgical intervention and emphasizes the crucial role of both intravenous antifungal therapy and surgical drainage in its treatment.

## Introduction

Fungal pericarditis is typically associated with post-cardiothoracic surgery and immunocompromised individuals [1–3]. Recognition can be challenging because of nonspecific clinical findings, nevertheless, timely administration of antifungal medications and prompt surgical drainage are crucial for effective management [3]. Most reported cases of fungal pericarditis are precipitated by cardiothoracic surgery but this case emphasizes the importance of recognizing the presentation in the absence of prior surgical intervention.

## Case report

A 40-year-old female with a history of IV drug use and liver dysfunction presented with MRSA bacteremia and spontaneous bacterial peritonitis. Despite intravenous antibiotics and supportive measures, the patient’s clinical status worsened eventually proceeding to a pulseless electrical activity arrest. Following successful resuscitation and transfer to high acuity care, cardiac echocardiography (ECHO) revealed significant mitral and tricuspid valve regurgitation suggestive of infective endocarditis (IE). Unfortunately, the patient developed both kidney and liver failure and these factors along with a poor clinical status precluded surgical intervention at that time. Despite broad-spectrum intravenous antibiotics the patient remained persistently febrile with an elevated leukocyte count. Further analysis of blood cultures identified gross candida and addition of IV antifungals led to a marked clinical improvement. Despite this, the patient again worsened and cardiac ECHO revealed a loculated pericardial effusion, with significant compression of the right atrium and both ventricles, suggestive of cardiac tamponade (Fig. 2). The patient was promptly taken to the operating room, a pericardial window made and 450 cc of purulent fluid drained (analysis confirmed the presence of candida), the pericardium was washed with warm sterile water and a 20-French chest tube placed. Intravenous antibiotics and anti-fungal medications were administered thereafter.

**Figure 1:**
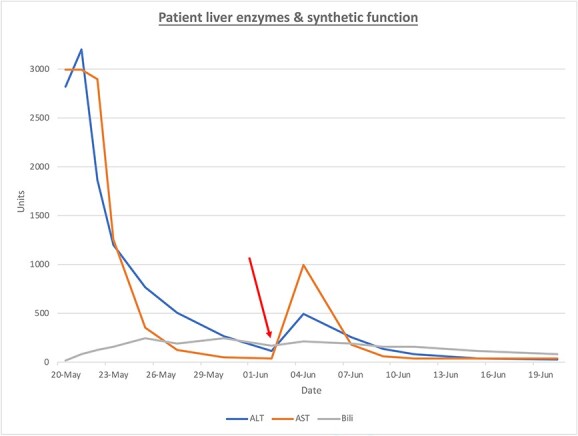
ALT (blue), AST (orange), Bili (bilirubin, gray) are presented over time. The red arrow represents the intervention of surgical drainage.

The patient’s clinical status drastically improved following surgical intervention, with progressively improving liver enzymes (Fig. 1). Following an extended period of clinical stability, the patient was transferred to a lower acuity setting. Unfortunately, the patient’s condition rapidly declined and with requested attenuation of both medical and surgical interventions, the patient expired.

**Figure 2:**
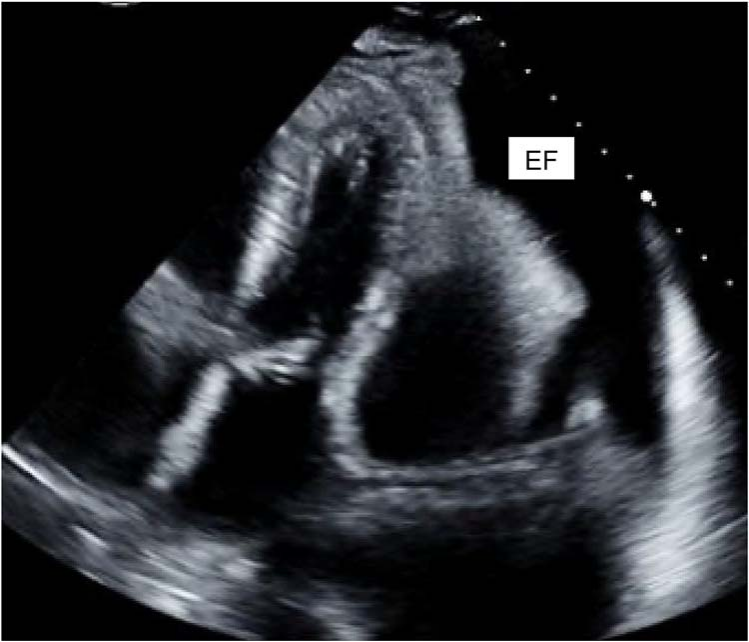
Cardiac ECHO images: ECHO images of the patient’s heart. EF represents effusion.

## Discussion

Fungal pericarditis is reported as a rare entity, thought to be related to 1% of cases of pericarditis, candida being the prototypical pathogen [1, 2]. However, the prevalence of fungal pericarditis may be underestimated. Postmortem autopsy findings across several studies of post-cardiac transplant patients revealed that the presence of candida which is most commonly found in the myocardium was present in a 30%–60% cases [3–5].

Candida-related pericarditis is thought to be caused by systemic colonization, or direct seeding following cardiac transplant or mediastinal infection [6, 7]. Candida colonization has been linked to renal insufficiency, surgical intervention, pancreatitis, mechanical ventilation, hemodialysis, and the use broad spectrum antibiotics [8], several of these risk factors being present in this case. Moreover, known risk factors for the development of fungal pericarditis include recent thoracic/abdominal surgery, cardiac transplantation, and immunosuppressive states induced by malignancy, chronic steroid use as well as sepsis and gross candida colonization [9–12]. Fungal pericarditis, like candida colonization, is related to the use of broad-spectrum antibiotics as they appear to promote fungal colonization of several organ systems, inducing a high burden of fungal load [8]. We suspect that the IE along with concomitant liver disease and use of broad-spectrum antibiotics contributed to the development of systemic fungal colonization and the resultant fungal pericarditis in this case. An alternative hypothesis is that the translocation of yeast from the mitral and tricuspid valves related to IE was the primary etiology. The patient’s cardiac function did marginally improve on antibiotics on initial presentation suggesting that a fungal pathogen was not the predominant etiology effecting cardiac output.

Typically, the presentation of candida pericarditis is nonspecific. However, a consistent and unexplained fever along with cardiac tamponade symptoms is certainly suggestive, in the context of cardiac and/or thoracic surgery, systemic colonization, etc. According to case report series, candida pericarditis is lethal unless promptly recognized and treated, the use of antifungal agents and operative drainage producing the best chance for cure [13]. Unfortunately, even with prompt recognition and surgical management, mortality is high [13, 14]. Additionally, Pericardiocentesis has been shown to temporize tamponade symptoms; however, a rapid re-accumulation of fluid is common and surgical drainage is the preferred method of definitive management [13].

This case serves as a reminder of the somewhat nonspecific and insidious presentation of fungal pericarditis. Mortality is sure without prompt recognition and intervention, and the utility of cardiac echo and clinical exam cannot be overstated in this regard. Moreover, surgical drainage and utilization of antifungal medications is the mainstay of treatment and gives the best chance for cure.

## Conflict of interest statement

None declared.

## Funding

None declared.
